# Gut Microbiome and ADHD: A Narrative Review of Clinical Evidence and Practice Implications

**DOI:** 10.1111/jhn.70327

**Published:** 2026-08-02

**Authors:** Kinga Sadowska, Kathryn Hart

**Affiliations:** ^1^ University of Surrey Guildford UK

**Keywords:** ADHD, adjunctive therapy, gut microbiome, gut‐brain axis, probiotics, synbiotics

## Abstract

**Objective:** This narrative review evaluates the current evidence on the efficacy of probiotic interventions for Attention Deficit Hyperactivity Disorder (ADHD) symptoms in both medicated and drug‐naïve paediatric and adult populations and assesses the implications for clinical dietetic practice.

**Design:** A narrative review synthesizing randomized controlled trials and observational microbiome studies in paediatric and adult populations, specifically distinguishing between probiotic monotherapy and adjunctive protocols.

**Results:** Observational data confirm gut microbiome alterations in ADHD populations, although specific bacterial signatures vary across studies. Evidence from treatment trials demonstrates that the efficacy of probiotics as monotherapy for core ADHD symptoms remains inconclusive. However, specific adjunctive trials combining probiotics with conventional medication have reported preliminary positive findings on symptom reduction, though results remain heterogeneous. Adult evidence is sparse but indicates potential benefits for emotional dysregulation in specific contexts.

**Conclusions:** This review concludes that current data do not support universal probiotic supplementation or routine clinical recommendation. However, when families inquire about complementary approaches, the existing literature enables evidence informed guidance within a shared decision‐making framework that acknowledges the preliminary nature of current findings and sets realistic expectations.

## Key Points

- Current evidence does not support the use of probiotics as a standalone treatment for core ADHD symptoms; therefore, they should not replace standard pharmacological care.
- Preliminary findings suggest that specific probiotic strains may offer symptomatic benefits when used adjunctively with medication, though results vary by strain and individual.
- Clinicians should utilize a shared decision‐making framework to help families interpret emerging evidence, ensuring that any complementary use is safe and that expectations are realistic.

## 1 Introduction

Attention deficit hyperactivity disorder (ADHD) is a prevalent neurodevelopmental condition affecting populations across the lifespan. The disorder is characterized by persistent patterns of inattention and hyperactivity‐impulsivity that directly impact academic, occupational, and social functioning [1]. Approximately 8% of children and adolescents and 3%–7% of adults worldwide meet diagnostic criteria for ADHD [2, 3, 4], with individuals experiencing significant functional impairment across multiple life domains [1].

Current management relies primarily on stimulant medications targeting dopaminergic neurotransmission [5]. While approximately 60% of patients demonstrate moderate‐to‐marked symptom improvement [5], concerns regarding side effects, treatment acceptability, and access disparities persist [6]. These limitations have prompted growing interest in complementary therapeutic strategies, with families increasingly seeking nutritional and lifestyle interventions to support standard care.

Recent research has investigated the gut microbiome as a potential therapeutic target through the microbiota‐gut‐brain axis. The theoretical rationale builds on bidirectional communication between the gastrointestinal tract and central nervous system through neural, endocrine, immune, and metabolic pathways [7, 8, 9]. Specifically, gut microbial production of neurotransmitter precursors, short‐chain fatty acids, and bioactive metabolites may influence the dopaminergic and inflammatory dysregulation characteristic of ADHD pathophysiology [10, 11, 12, 13]. Foundational studies demonstrate that specific probiotic strains can modulate central neurotransmitter receptor expression [14], with evidence showing beneficial effects on anxiety and stress‐related outcomes [15].

Given the growing research interest in the microbiota‐gut‐brain axis and the increasing number of families inquiring about complementary approaches, critical evaluation of the clinical evidence is warranted. This narrative review synthesizes findings from randomized controlled trials in paediatric and adult populations alongside observational microbiome evidence. This review distinguishes between monotherapy and adjunctive use to examine whether current data can inform dietetic practice when families seek guidance on probiotic interventions.

## 2 Methods

This narrative review was conducted following the SANRA (Scale for the Assessment of Narrative Review Articles) guidelines to enhance methodological transparency and quality [16]. PubMed/MEDLINE, Embase, PsycINFO, and Google Scholar were searched between January 2000 and October 2025, combining terms related to ADHD (“attention deficit hyperactivity disorder” OR “ADHD”), microbiome interventions (“probiotic*” OR “synbiotic*” OR “microbiome” OR “gut microbiota”). Searches were limited to peer‐reviewed English‐language articles. Reference lists of included systematic reviews and meta‐analyses were hand‐searched to identify additional studies.

Included study designs comprised randomized controlled trials, systematic reviews, meta‐analyses, and observational studies documenting microbiome alterations in ADHD populations. Studies were excluded if they focused solely on other neurodevelopmental conditions or involved only non‐human subjects. To capture theoretical context for evaluating intervention rationale, foundational studies on gut‐brain axis mechanisms were included [7, 8] and comprehensive reviews on gut microbiota in neurodevelopmental disorders [9, 11], identified through targeted citation searching.

## 3 Observational Evidence

Before evaluating whether probiotics help, we need to ask: do people with ADHD actually have different gut microbiomes? If not, there would be little reason to expect microbiome‐targeted interventions to work.

The evidence suggests yes, though findings are inconsistent. Evidence comes from cross‐sectional case‐control studies, longitudinal birth cohorts, and systematic reviews.

Pediatric studies yield mixed findings. Investigations have reported decreased [17], increased [18], or no significant differences [19] in gut bacterial diversity between children with ADHD and healthy controls. One study of 14 boys with ADHD found reduced bacterial variety compared to 17 controls [17]. Another, with 30 children, found higher diversity in the ADHD group [18]. A third, comparing 41 children with ADHD to 39 controls, found no differences [19].

These inconsistencies persist despite similar sequencing methods (16S rRNA), suggesting other factors play a role: age, sample size, diet, medication status, and geography. Nevertheless, several bacterial genera appear repeatedly as altered in ADHD, including *Faecalibacterium* [20, 21], *Bifidobacterium* [20, 22], *Ruminococcus* [23, 24], and *Bacteroides* [18, 21].

Longitudinal birth cohort studies offer stronger evidence by tracking children over time. The WHEALS cohort (*n* = 314) collected stool samples at 1 and 6 months and assessed ADHD at age 10 [25]. Of these children, 59 (18.8%) developed ADHD. At 6 months, bacterial diversity was significantly higher in children who later developed ADHD (*p *= 0.017), and bacterial composition differed between groups (*p *= 0.006). Notably, children who developed ADHD showed relative depletion of lactic acid bacteria, with 14 taxa from the order *Lactobacillales* underrepresented. Fungal microbiota also differed between groups at both time points.

A Swedish cohort tracking 16,440 children found similar patterns, identifying early microbiome signatures among biomarkers predicting neurodevelopmental disorders [26]. These prospective studies provide the strongest observational support for a potential causal link.

Adult studies show similar patterns. A meta‐analysis of four case‐control studies (312 adults with ADHD, 305 controls) found consistent alterations in gut microbiome composition, including increased *Ruminococcus torques* and decreased *Eubacterium xylanophilum* [23]. Systematic reviews note that some taxa replicate across studies, particularly decreased *Faecalibacterium prausnitzii*, but methodological variation prevents definitive conclusions [20, 21, 27, 28].

Some findings point in opposite directions. *Bifidobacterium* appears elevated in some populations but reduced in others. *Dialister* is decreased in unmedicated patients but increased after treatment [28].

**Figure 1:**
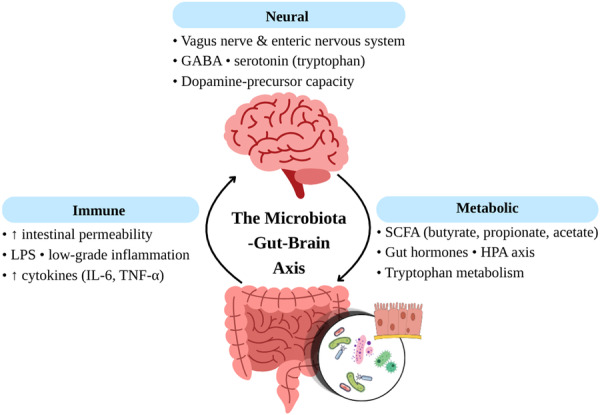
Three principal pathways of the microbiota‐gut‐brain axis implicated in ADHD. **Neural**: vagus nerve and enteric nervous system signalling modulates central GABA, serotonin, and dopaminergic systems [9, 14]. **Immune**: increased intestinal permeability and microbial products (e.g., lipopolysaccharide) drive low‐grade inflammation and cytokine signalling [8, 13]. **Metabolic**: bacterial short‐chain fatty acids and microbially shaped tryptophan metabolism influence microglia, the blood‐brain barrier, and neurotransmission [10, 33]. The pathways overlap rather than act independently [29]. *Abbreviations:* ADHD ‐ attention deficit hyperactivity disorder; GABA ‐ gamma‐aminobutyric acid; HPA ‐ hypothalamic‐pituitary‐adrenal; IL ‐ interleukin; LPS ‐ lipopolysaccharide; TNF ‐ tumour necrosis factor.

The studies above establish that the ADHD microbiome differs from controls, but correlation alone says nothing about how these differences could shape behaviour. The plausibility of microbiome‐targeted intervention rests on the routes through which gut microbes can influence the brain. Three principal pathways are most relevant to ADHD and are described below, although they overlap rather than act independently (Figure 1) [29].

The first pathway is neural. The vagus nerve and enteric nervous system provide direct bidirectional communication between gut and brain, and microbes can signal through this route via the production of neurotransmitters and their precursors to alter central neurotransmission. Several of the genera altered in ADHD contribute here. For example, *Lactobacillus* and *Bifidobacterium* synthesise gamma‐aminobutyric acid (GABA) and influence the availability of tryptophan for serotonin synthesis. Lactic‐acid‐producing genera are depleted in several paediatric cohorts [20, 25], whereas the direction for *Bifidobacterium* is inconsistent, with reductions in some infant cohorts but increases in adolescent and adult ADHD [28]. Most relevant to ADHD, the microbiota can also act on dopaminergic and wider catecholaminergic neurotransmission, the systems central to the disorder [9, 20, 30]; for instance, the increased *Bifidobacterium* abundance reported in ADHD has been associated with greater microbial capacity for synthesis of a dopamine precursor [28]. In a preclinical model, a *Lactobacillus* strain modified central GABA‐receptor expression and behaviour, and the effect was abolished by vagotomy [14].

The second pathway is immune. Microbial alterations and increased intestinal permeability may allow products such as lipopolysaccharide to enter the circulation, promoting low‐grade inflammation and cytokine signalling that can act on the brain [8]. Frequently reported, though not consistently replicated across cohorts, is the depletion of the butyrate‐producing *Faecalibacterium prausnitzii* [20, 21], which normally supports gut‐barrier integrity and exerts anti‐inflammatory effects, alongside enrichment of taxa such as *Ruminococcus* that have been linked to inflammation [23, 24]. Elevated pro‐inflammatory cytokines (e.g., IL‐6, TNF‐α) have been proposed to impair the blood‐brain barrier (BBB) and act on the prefrontal and striatal circuits implicated in ADHD [30]. In ADHD this cascade remains proposed rather than established, but it aligns with evidence implicating inflammation in the disorder [13].

The last pathway is metabolic. Bacterial fermentation produces short‐chain fatty acids (SCFAs), principally butyrate, propionate, and acetate, which signal along the axis and influence microglia, the blood‐brain barrier, and neurotransmission [10]. Beyond crossing the blood‐brain barrier, SCFAs activate free fatty acid receptors on enteroendocrine cells, modulating gut‐hormone release and hypothalamic‐pituitary‐adrenal (HPA) axis activity [31]. Notably, lower faecal SCFA concentrations have been reported in psychostimulant‐medicated than in unmedicated children with ADHD [32], linking this pathway to the medication‐status differences discussed later. The microbiota also shapes tryptophan metabolism, shifting it toward either the serotonin or the kynurenine route [33]. Both systems are implicated in ADHD [9].

These three pathways do not operate in isolation but form an interconnected network. For example, SCFA deficiency simultaneously compromises BBB integrity (metabolite pathway), reduces anti‐inflammatory signaling (immune pathway), and alters vagal afferent signaling (neural pathway). Similarly, dysbiosis‐driven inflammation can impair vagal tone, which in turn worsens gut barrier function [29, 30]. It is important to note that while the evidence linking these pathways to ADHD is growing, much of it remains correlational, and results across studies are heterogeneous [29].

## 4 Probiotic Interventions: Evidence from Clinical Trials

Having established biological plausibility, the following sections examine whether intervention trials support a therapeutic role for probiotics in ADHD. To answer this, we reviewed 13 clinical trials alongside three systematic reviews and meta‐analyses [34, 35, 36]. Collectively, the 13 trials comprised approximately 800 participants across Europe, Asia, the Middle East, and North America. Most trials were small (30 to 182 participants) and short (8 to 12 weeks). Probiotic strains, doses, and outcome measures varied considerably. The three reviews stratify the current evidence base by developmental stage, with two focusing on pediatric populations [34, 35] and one examining interventions in adults [36].

We also considered observational evidence: cross‐sectional microbiome studies [19, 22, 37], birth cohort investigations linking infant microbiome patterns to later ADHD [25, 26], systematic reviews of microbiome findings [20, 21, 27, 28], and one case report of symptom improvement after fecal microbiota transplantation [38].

We organize the trials by treatment context. The pediatric evidence comprised ten treatment studies (ages 4–18 years) and one prevention study following infants for 13 years, with two further trials in adults. Table 1 summarizes trial characteristics and outcomes. The sections below address early‐life prevention, monotherapy in drug‐naïve populations, and adjunctive therapy alongside medication.

**Table 1:** Summary of Studies on the Association Between ADHD and Probiotics/Prebiotics.

Study	Country	Age	${\;n\;}_{p}$	${\;n\;}_{c}$	Probiotic (dosage)	Rx	Design	Weeks	Outcome
Pärtty 2015	Finland	0–13^o^	40	35	*L. rhamnosus* GG (1 $\times 10^{10}$ CFU/day)^a^	None	DB‐RCT	26	0% ADHD/AS vs 17% placebo (*p* = 0.008)
Kumperscak 2020	Slovenia	4–17	18	14	*L. rhamnosus* GG ($\geq 1\times 10^{10}$ CFU/day)^b^	None	DB‐RCT	12	Improved QoL (*p* = 0.044); no symptom change
Wang 2022	Taiwan	4–16	30	—	*B. bifidum* Bf‐688 (5 $\times 10^{9}$ CFU/day)^c^	None	Open	8	Improved inattention and H/I (open‐label)
Bazinet 2017	Canada	6–15	25	23	*L. helveticus* R0052 + *B. longum* R0175 (3 $\times 10^{9}$ CFU/day)^d^	Mixed	DB‐RCT	4	No change (primary); improved in non‐medicated subgroup
Skott 2020^n^	Sweden	5–18	42	26	Synbiotic 2000 (4 $\times 10^{11}$ CFU/day)^e^	Mixed	DB‐RCT	9	No ADHD change; reduced RRB (p<0.05)
Yang 2023^n^	Sweden	8–18	28	21	Synbiotic 2000 (4 $\times 10^{11}$ CFU/day)^e^	Mixed	DB‐RCT	9	Reduced IL‐12/IL‐23p40 (*p* = 0.020); increased propionic acid (*p* = 0.046)
Rojo‐Marticella 2025	Spain	5–16	20	18	*Lactiplantibacillus plantarum* CECT7485 + *Levilactobacillus brevis* CECT7480 (1 $\times 10^{9}$ CFU/day)^f^	Mixed	DB‐RCT	12	Improved H/I in age 5–9y (d = 0.692)
Sepehrmanesh 2021	Iran	8–12	17	17	*L. reuteri, L. acidophilus, L. fermentum, B. bifidum* (8 $\times 10^{9}$ CFU/day)^g^	Ritalin	DB‐RCT	8	Improved ADHD‐RS (*p* = 0.006), inattention (*p* = 0.009), H/I (*p* = 0.03)
Ghanaatgar 2023	Iran	6–12	21	17	Bio‐Kult 14 strains (2 $\times 10^{9}$ CFU/day)^h^	Ritalin	DB‐RCT	8	Improved CPRS‐RS wk 4 (*p* = 0.014) & 8 (p<0.001); lower CGI‐S (*p* = 0.018)
Sangsefidi 2025	Iran	4–16	30	30	*L. plantarum* A7 + *B. animalis* BB‐12 (3.5 $\times 10^{7}$–3.5 $\times 10^{8}$ CFU/day)^i^	Ritalin	TB‐RCT	8	Decreased CPRS wk 4 (*p* = 0.01); increased auditory control (*p* = 0.02)
Elhossiny 2023	Egypt	6–16	36	40	*L. acidophilus* LB (4 $\times 10^{10}$ CFU/day)^j^	Atomox.	Open RCT	12	Improved inattention (*p* = 0.007), H/I (*p* = 0.035), CBCL
Wang 2024	Taiwan	6–12	51	51	*B. bifidum* Bf‐688 (5 $\times 10^{9}$ CFU/day)^k^	MPH	DB‐RCT	12	No symptom change; improved CPT performance
Levy 2024	Israel	19–30	30	30	*L. helveticus, B. animalis, E. faecium, B. longum, B. subtilis* (4 $\times 10^{10}$ CFU/day)^l^	None	DB‐RCT	12	Reduced hyperactivity (*p* = 0.012); improved academic grades
Arteaga‐Henríquez 2024	Hungary, Spain, Germany	18–65	56	57	Synbiotic 2000 Forte (4 $\times 10^{11}$ CFU/day)^m^	Mixed	DB‐RCT	10	17% vs 4% response (*p* = 0.01); improved irritability, inattention

*Note:* CFU/day totals are derived from manufacturer label specifications reported in each paper. The exception is Sangsefidi 2025, where the probiotics were custom‐encapsulated for the trial in alginate pearls, with a target viability of $10^{6}-10^{7}$ CFU. For multi‐strain products, the total reflects the per‐strain count summed across strains and multiplied by daily dose frequency. Dose details per source paper. ^n^p = probiotic sample; ^n^c = control sample; Rx = pharmacological treatment; Atomox.=Atomoxetine; DB‐RCT = Double‐blind randomized controlled trial; TB‐RCT = Triple‐blind randomized controlled trial; Open = Open‐label trial; H/I = hyperactivity/impulsivity; RRB = restricted/repetitive behaviors; wk = week; MPH = Methylphenidate; CFU = Colony Forming Units; QoL = quality of life; CPRS‐RS = Conners Parent Rating Scale; CGI‐S = Clinical Global Impression–Severity; CPT = continuous performance test; ADHD‐RS = ADHD Rating Scale; CBCL = Child Behavior Checklist.

^a^ 1 capsule daily; 1 $\times 10^{10}$ CFU per capsule.

^b^ 1 capsule daily; $\geq 10^{10}$ CFU per capsule.

^c^ 2 sachets daily (morning + evening); 5 $\times 10^{9}$ CFU total per day.

^d^ 1 Probiotic Stick (Lallemand) packet daily; 3 $\times 10^{9}$ CFU combined per packet.

^e^ 1 sachet daily; 4 $\times 10^{11}$ CFU per sachet of three lactic acid bacteria.

^f^ 1 sachet daily; 1 $\times 10^{9}$ CFU per sachet (two strains 1:1).

^g^ 1 sachet daily; 8 $\times 10^{9}$ CFU total (4 strains, 2 $\times 10^{9}$ each).

^h^ 1 capsule daily; 2 $\times 10^{9}$ CFU per capsule (Bio‐Kult, 14 strains).

^i^ 35 alginate pearls daily; $10^{6}-10^{7}$ CFU per pearl (two strains 1:1).

^j^ 4 sachets daily (2 sachets × 2 doses); $10^{10}$ CFU per sachet.

^k^ 2 sachets daily (morning + evening); 5 $\times 10^{9}$ CFU total per day.

^l^ 2 capsules once daily; 8 $\times 10^{9}$ CFU per strain × 5 strains.

^m^ 1 sachet daily; 4 $\times 10^{11}$ CFU per dose (Synbiotic 2000 Forte).

^n^ Same underlying RCT reported across two papers; Skott 2020 reports primary outcomes from the full children cohort; Yang 2023 reports IL‐12/IL‐23p40 and SCFA outcomes from the subset that provided blood samples.

o Intervention 0–6 months; outcomes at 13 years.

### 4.1 Early‐Life Prevention

Only one trial examined whether early‐life probiotics might prevent ADHD. Pärtty et al. randomized 159 infants to receive *Lactobacillus rhamnosus* GG or placebo during the first 6 months of life [39]. Of the 75 children who completed 13‐year follow‐up (40 probiotic, 35 placebo), none in the probiotic group developed ADHD or Asperger syndrome, compared to 17.1% in the placebo group (*p *= 0.008). Affected children had lower infant *Bifidobacterium* levels. This finding aligns with prospective cohort evidence linking early microbiome patterns to later ADHD [25, 26].

### 4.2 Monotherapy in Drug‐Naïve Populations

Only two trials examined probiotics as standalone treatment in children not taking ADHD medication. Kumperscak et al. conducted a double‐blind, placebo‐controlled trial of *Lactobacillus rhamnosus* GG in 32 children over 12 weeks [40]. Children in the probiotic group reported significant improvements in health‐related quality of life compared to placebo (*p* = 0.044). However, parent‐reported ADHD symptoms improved in both groups with no between‐group difference. Teacher‐reported symptoms showed no significant changes.

Wang et al. took a different approach, using an open‐label, single‐arm design to test *Bifidobacterium bifidum* Bf‐688 in 30 drug‐naive children over 8 weeks [41]. Compared to baseline, children showed improvements in parent‐ and teacher‐rated symptoms (SNAP‐IV), along with weight gain and shifts in gut microbiota composition. The absence of a placebo group and blinding limits what can be concluded from these findings. Together, these two trials suggest that the effectiveness of probiotic monotherapy for core ADHD symptoms remains inconclusive.

### 4.3 Adjunctive Therapy With Pharmacological Treatment

Most trials examined probiotics alongside standard ADHD medication. Results were mixed, but some patterns emerged. We organize these findings by medication type.

Elhossiny et al. investigated *Lactobacillus acidophilus* LB as an add‐on to atomoxetine in 76 children [42]. The study used an open‐label design with blinded outcome assessors. Compared to the atomoxetine‐only group (*n* = 40), the probiotic group (n = 36) showed significant improvements in parent‐reported ADHD symptoms (inattention, hyperactivity‐impulsivity, and total scores; p<0.05), attention measures (target accuracy: *p* = 0.02; omission errors: *p* = 0.043), and executive function (perseverative errors: *p* = 0.017; non‐perseverative errors: *p* = 0.044).

Four trials combined probiotics with methylphenidate (Ritalin). The largest was Wang et al.'s double‐blind placebo‐controlled trial in 102 children on stable methylphenidate [43]. The *Bifidobacterium bifidum* Bf‐688 group showed significant improvements in computerized performance test measures (omission errors, reaction time). However, parent‐reported symptoms improved in both groups with no additional benefit from the probiotic.

Three smaller trials reported more positive findings. Sepehrmanesh et al. tested a four‐strain formulation (*Lactobacillus reuteri*, *L. acidophilus*, *L. fermentum*, *Bifidobacterium bifidum*) in 34 children aged 8–12 years [44]. The probiotic group showed improvements in parent‐rated total symptoms (*p* = 0.006), inattention (*p* = 0.009), hyperactivity‐impulsivity (*p* = 0.03), and clinician‐rated anxiety (*p* = 0.01), along with improved inflammatory markers and antioxidant capacity.

Ghanaatgar et al. conducted a double‐blind trial in 38 children aged 6–12 years [45]. After 8 weeks, the probiotic group showed an 18.6‐unit reduction in Conners Parent Rating Scale scores compared to placebo (p<0.001) and lower Clinical Global Impression‐Severity scores (0.7‐unit difference, *p* = 0.018).

Sangsefidi et al. tested a two‐strain formulation (*Lactobacillus plantarum*, *Bifidobacterium animalis*) in 60 children [46]. Symptom improvements appeared at 4 weeks but were not sustained at 8 weeks. The probiotic group did show higher auditory response control scores at endpoint (91.55 ± 16.69 vs. 80.55 ± 17.43; *p* = 0.02).

Four trials enrolled children regardless of medication use, making it harder to isolate adjunctive effects.

Skott et al. examined Synbiotic 2000 for 9 weeks in 68 children aged 5–18 years [47]. Core ADHD symptoms and functioning did not differ significantly between the synbiotic and placebo treatment groups. However, exploratory analyses revealed reduced autistic traits specifically in the synbiotic group, with effects driven by children with elevated baseline sVCAM‐1 (a vascular inflammation marker) and those unmedicated. A secondary analysis by Yang et al. examined biological markers in the same trial [48]. Compared to placebo, the synbiotic significantly reduced inflammatory markers (IL‐12/IL‐23p40; *p* = 0.007) in medicated children (n = 32) and increased propionic acid (*p* = 0.046) in all participants (n = 49).

Bazinet et al. tested *Lactobacillus helveticus* R0052 and *Bifidobacterium longum* R0175 over 28 days in 48 children with ADHD and/or anxiety [49]. In the full sample, the probiotic group showed improved visual memory (*p* = 0.001, partial $\eta^{2}=0.229$) but no change in parent‐reported symptoms (*p* = 0.90). A subsample analysis excluding children on stimulants (n = 36) found probiotic benefits for parent‐reported hyperactivity (*p* = 0.04) and both verbal (*p* = 0.03) and visual memory (*p* = 0.001), though not for inattention or overall symptoms.

Rojo‐Marticella et al. investigated *Lactiplantibacillus plantarum* and *Levilactobacillus brevis* for 12 weeks in children with comorbid ASD and ADHD [50]. In younger children (5–9 years) with ADHD, probiotics showed a moderate‐to‐large effect on hyperactivity‐impulsivity versus placebo that did not reach significance (*p* = 0.181, d = 0.692), though within‐group improvement from baseline was significant (*p* = 0.016, d = −1.030).

### 4.4 Adult Intervention Evidence

Adult evidence is sparse. Only two randomized controlled trials and one case report address this population.

Levy et al. conducted a 3‐month double‐blind trial in 60 college students (aged 19–30) with ADHD [51]. Participants received either a multi‐strain probiotic (*L. helveticus*, *B. animalis* ssp. *lactis*, *Enterococcus faecium*, *B. longum*, and *Bacillus subtilis*) or placebo. The probiotic group showed significant within‐group reductions in hyperactivity symptoms on the MOXO computerized performance test (*p* = 0.012), though between‐group comparisons were not reported. Academic performance improved within the probiotic group (*p* = 0.004) but not compared to placebo (*p* = 0.709). Gastrointestinal symptoms decreased in the probiotic group (*p* = 0.007) with no change in the placebo group.

The PROBIA trial examined synbiotic supplementation in 180 adults with ADHD and/or borderline personality disorder over 10 weeks (113 completed the trial) [52]. This double‐blind, placebo‐controlled study defined response as at least 30% reduction in self‐reported irritability plus clinician‐rated improvement. The synbiotic group showed a higher responder rate than placebo (17% vs 4%; *p* = 0.01) and significant improvements in emotional dysregulation (*p* = 0.03), inattention (*p* = 0.01), functioning (*p* = 0.03), and perceived stress (*p* = 0.03).

A systematic review by Gomes et al. underscores how limited this evidence base remains: of 3,591 studies screened, only three randomized controlled trials met inclusion criteria [36].

One case report documented ADHD symptom improvement following fecal microbiota transplantation in a 22‐year‐old woman treated for recurrent *Clostridioides difficile* infection [38]. Successful engraftment included species previously associated with lower ADHD risk, such as *Faecalibacterium prausnitzii*. No studies have systematically investigated FMT for ADHD.

## 5 Interpreting Mixed Findings

The previous section reviewed 13 trials. Results were mixed, some showed benefits, others did not. Before drawing conclusions, we need to understand why findings varied so much, and whether any consistent patterns emerge.

Methodological differences matter, and the two monotherapy trials illustrate this well. The blinded, placebo‐controlled trial found no difference in parent‐ or teacher‐reported symptoms, though children's self‐reported quality of life improved [40]. The open‐label trial reported symptom improvements [41], but without blinding or a control group, these could reflect natural fluctuation or placebo response.

Protocol heterogeneity complicates comparison. Trials varied in probiotic strains, dosing, and duration. This makes synthesis difficult and rules out any “one‐size‐fits‐all” recommendation.

Medication status also appears relevant. Results were notably less consistent in populations with mixed medication status, where participants used various medications or none. In these groups, interventions generally failed to improve core symptoms [47, 49]. However, biological changes were still detectable: synbiotic supplementation reduced inflammatory markers (IL‐12/IL‐23p40) in medicated subgroups [48].

Despite the variability, one pattern emerges: probiotics may work better alongside standard medication than alone.

Several trials combining probiotics with methylphenidate or atomoxetine reported symptom improvements [42, 44, 45]. Multi‐strain formulations containing *L. reuteri* and *Bifidobacterium* species, or *L. acidophilus* adjuncts, showed superior symptom reduction compared to medication alone in some studies. Meta‐analyses support this pattern, reporting larger effect sizes for adjunctive versus monotherapy approaches [34, 35].

Several mechanisms could explain this pattern. The gut microbiome may influence medication metabolism, or probiotics and pharmacotherapy may act on complementary pathways. However, these possibilities remain speculative and require mechanistic investigation.

The pattern is not universal. Some adjunctive trials found benefits only in specific domains, computerized attention tests or auditory control, without changes in parent‐reported behavior [43, 46]. Notably, the largest adjunctive trial (*n* = 102) found no additional benefit on parent‐reported symptoms [43].

Adult evidence, though sparse, fits this pattern. Synbiotics showed a higher clinical response rate than placebo in adults with ADHD and emotional dysregulation [52].

These findings must be interpreted cautiously given several limitations of the current evidence base. Most trials enrolled 30–70 participants and lasted 8–12 weeks. These small samples limit statistical power, and the short durations leave open whether benefits persist, fade, or emerge over longer periods.

Prevention data are preliminary. One trial observed no ADHD cases in the probiotic group versus 17% in placebo; however, this was a post‐hoc analysis of a small sample [39]. Independent replication is essential before any prevention claims can be made.

Outcome measures varied widely. Trials used different rating scales and different raters, parents, teachers, clinicians, children themselves. This limits cross‐study comparison and weakens meta‐analytic synthesis.

Taken together, the evidence does not support probiotics as a replacement for standard ADHD treatment. What it does suggest is that adjunctive use warrants further investigation, ideally with standardized protocols, larger samples, and longer follow‐up.

## 6 Implications for Clinical Practice

These findings have practical implications for dietitians and clinicians encountering families who inquire about complementary approaches.

Current evidence does not support universal probiotic supplementation for ADHD, not for prevention, and not as standalone treatment. The variability in strains, dosing, and study design makes any general prescription premature.

When families do ask about complementary approaches, probiotics may come up, they are widely available. In such cases, clinicians can offer evidence‐informed guidance rather than dismissal or endorsement.

The key message: probiotics might have a role alongside medication, not instead of it. Some adjunctive trials have shown benefit, particularly multi‐strain formulations containing *L. reuteri*, *Bifidobacterium* species, or *L. acidophilus* [42, 44, 45]. These findings are preliminary and do not constitute strain‐specific recommendations, but they provide a basis for informed discussion.

Safety profiles across reviewed trials were favorable, with no serious adverse events reported [34]. This allows for cautious openness rather than blanket caution.

In practice, this means framing probiotics as an area of active investigation where some trials show promise but definitive answers remain elusive. Families should understand that any probiotic use would complement, not replace, evidence‐based pharmacological and behavioral therapies. Expectations should be realistic: modest benefits in some individuals, no benefit in others, and no way yet to predict who might respond.

Shared decision‐making is the appropriate framework. Clinicians can acknowledge the preliminary nature of findings, highlight the adjunctive pattern in the literature, and support families in making informed choices that align with their values and circumstances.

## 7 Conclusion and Future Directions

This review confirms that gut microbiome alterations are a documented feature of ADHD, but translating these biological insights into effective interventions remains unclear. The evidence suggests a distinction: probiotics as monotherapy lack support, while some adjunctive trials have reported preliminary benefits when combined with conventional medication.

For clinical practice, this evidence does not warrant routine recommendation. However, when families inquire, clinicians can frame probiotics as an area of active investigation, some trials show promise, but definitive conclusions remain elusive. Any use should complement, not replace, evidence‐based care.

This synthesis, as a narrative review, introduces potential selection bias and limits reproducibility as it does not adhere to a formal systematic protocol. The heterogeneity of the studies reviewed, varying in strains, dosing, and outcome measures, precludes definitive conclusions for many outcomes. Publication bias against negative results must also be acknowledged.

Several priorities emerge for future research. First, trials should prioritize adjunctive designs, building on the pattern observed in existing studies. Identifying baseline microbiome characteristics that predict responder status would help target interventions to those most likely to benefit. Second, study durations must extend beyond 12 weeks to assess whether effects persist, and sample sizes must increase to allow stratification by age and other relevant factors. Third, standardized outcome assessment using validated, rater‐blinded instruments would strengthen cross‐study comparison and enable meaningful meta‐analysis.

Finally, from a nutritional science perspective, future protocols must incorporate rigorous dietary assessment. Background diet profoundly modulates microbiome composition, yet most trials did not monitor intake, leaving open the possibility that observed effects were shaped by host dietary habits rather than supplementation alone. Validated tools such as food frequency questionnaires or repeated 24‐h recalls should become standard in this field.

## Author Contributions

Kinga Sadowska conducted the literature review, synthesized the findings and drafted the manuscript. Kathryn Hart provided critical guidance, contributed to the refinement of the review structure and revised the manuscript. Both authors reviewed and approved the final version of the manuscript.

## Funding

The authors have nothing to report.

## Conflicts of Interest

The authors declare no conflicts of interest.

## Data Availability

Data sharing is not applicable to this article as no new data were created or analysed in this study. Data sharing not applicable to this article as no datasets were generated or analysed during the current study.
